# Toward water-smart cereal production: a narrative review of agronomic, genetic, and digital innovations for water productivity and climate resilience

**DOI:** 10.3389/fpls.2026.1826562

**Published:** 2026-05-15

**Authors:** Mohamed Houssemeddine Sellami, Marie Therese Abi Saab, Rossella Albrizio

**Affiliations:** 1 Institute for Mediterranean Agricultural and Forestry Systems (ISAFOM), National Research Council of Italy (CNR), Naples, Italy; 2 Climate and Water Unit, Lebanese Agricultural Research Institute, Fanar, Lebanon

**Keywords:** Alternate wetting and drying, conservation agriculture, deficit irrigation, digital irrigation, drought-resilient varieties, genetics × agronomy × digital framework

## Abstract

Cereal crops consume more freshwater than any other category of agricultural production, yet no recent review has quantitatively integrated agronomic, genetic, and digital water-smart innovations across the seven cereals most relevant to global food security: wheat (*Triticum* spp.), rice (*Oryza sativa* L.), maize (*Zea mays* L.), barley (*Hordeum vulgare* L.), sorghum (*Sorghum bicolor* [L.] Moench), pearl millet (*Pennisetum glaucum* [L.] R.Br.), and finger millet (*Eleusine coracana* [L.] Gaertn.). This narrative review addresses that gap by synthesizing 110 studies — retrieved through a PRISMA-inspired selection workflow and appraised on a four-dimensional quality rubric — across five intervention domains: deficit irrigation, soil-moisture conservation, drought-resilient varieties, precision agriculture and digital irrigation, and integrated water management. Quantitative pooled findings indicate that mild alternate wetting and drying in rice reduces irrigation water by ~23% with negligible yield penalty, whereas severe drying incurs a ~23% yield loss; deficit irrigation in wheat improves water productivity by ~7% but reduces yield by ~16%; and straw mulching raises cereal yields by 13–22% (pooled). IoT-based scheduling delivers meaningful but heterogeneous irrigation-water savings relative to calendar-based practice, although the cereal-specific evidence base remains thin relative to horticultural applications. The review’s principal contribution is the Genetics × Agronomy × Digital (G×A×D) integration framework, which maps these three intervention families onto the soil–plant–atmosphere–institution continuum and makes their combinatorial opportunities and institutional prerequisites explicit. The central critical conclusion is that the binding constraint on water-smart cereal production has shifted from the technical feasibility of individual interventions to the institutional, financial, and governance capacity required to combine them equitably, particularly in smallholder systems. Evidence for finger millet and for rainfed smallholder systems in sub-Saharan Africa remains notably thin and represents a priority gap.

## 1 Introduction

Cereal crops provide more than half of the world’s direct calories and remain the essential feedstock for livestock production (Shiferaw et al., 2013; FAO, 2024). Wheat, rice, maize, barley, sorghum, pearl millet, and finger millet collectively occupy more than 700 million hectares of arable land, spanning tropical lowlands to temperate highlands and irrigated plains to rainfed drylands (Ray et al., 2013). Agriculture withdraws approximately 70% of global freshwater, and cereals are the single largest consumer within that allocation (Mekonnen and Hoekstra, 2011; Mialyk et al., 2024). Paddy rice alone uses two to five times more water per unit of grain than other cereals, while irrigated wheat and maize in arid and semi-arid regions increasingly deplete non-renewable groundwater (LaHue et al., 2016; Kumar and Rajitha, 2019).

Climate change is intensifying these pressures. Observed warming of 1.0–1.5 °C above pre-industrial levels has already accelerated the hydrological cycle, increased evapotranspiration, and shifted precipitation regimes across major cereal-growing regions (Zhao et al., 2017; IPCC, 2023). Each additional 1 °C of warming is projected to reduce global wheat yields by 6–7% and maize yields by 7–10%, with the most severe impacts concentrated in tropical and subtropical regions (Abdul et al., 2025; Tran et al., 2025). Water availability for agriculture is expected to decline by 10–30% by 2050 in critical river basins of South Asia, sub-Saharan Africa, and the Mediterranean (Burek et al., 2016).

In response, the concept of “water-smart” agriculture has emerged as a unifying paradigm that maximizes crop output per unit of water consumed through a portfolio of complementary interventions (Kijne et al., 2003; Frimpong et al., 2023). The portfolio spans conventional agronomic practices (deficit irrigation, residue mulching, conservation tillage), genetic improvement (conventional breeding, marker-assisted selection, genomic selection, gene editing), and digital tools (satellite remote sensing, IoT-based soil-moisture monitoring, AI-driven decision-support systems) (Ali et al., 2025; Muharomah et al., 2025).

Despite a rapidly expanding body of water-saving research, three gaps remain. First, existing reviews focus predominantly on single cereals (notably rice or wheat) or single intervention families (breeding, mulching, or sensors) and therefore do not quantify how these interventions interact across the seven major cereal species (Kang et al., 2017; Yamini et al., 2025). Second, few reviews integrate the agronomic, genetic, and digital literatures under a common conceptual framework. Third, most available syntheses describe technical potential but do not systematically account for socio-economic, institutional, and governance constraints on adoption. The present review addresses these three gaps by (i) synthesizing evidence across the seven cereals most relevant to global food security, (ii) proposing an explicit Genetics × Agronomy × Digital (G×A×D) integration framework, and (iii) embedding the technical analysis within a critical discussion of adoption barriers, equity, and basin-scale water-accounting consequences.

## 2 Materials and methods

### 2.1 Review design and rationale

This review adopts a narrative synthesis approach (Grant and Booth, 2009; Ferrari, 2015; Greenhalgh et al., 2018) to examine agronomic, genetic, and digital innovations that enhance water productivity. Narrative synthesis is particularly suited to topics spanning multiple disciplines, crop species, and methodological traditions, where the heterogeneity of study designs and outcome metrics precludes standardized quantitative pooling (Grant and Booth, 2009). This review addresses five thematic domains defined *a priori* from established water-smart frameworks (Kijne et al., 2003; Molden, 2013): (i) deficit irrigation, (ii) soil moisture conservation, (iii) drought-resilient varieties, (iv) precision agriculture and digital irrigation, and (v) integrated water management.

### 2.2 Search strategy

Literature retrieval was performed between January and March 2026 using four academic databases (Web of Science, Scopus, PubMed, Google Scholar) and institutional repositories of FAO, IWMI, ICRISAT, and the CGIAR system. Queries combined crop-specific terms (wheat, rice, maize, barley, sorghum, millet, cereal, and their Latin binomials) with domain-specific descriptors grouped by Boolean operators. Water-management terms included water productivity, deficit irrigation, alternate wetting and drying (AWD), soil-moisture conservation, mulching, precision irrigation, and water footprint. Technology terms included remote sensing, IoT, precision agriculture, artificial intelligence, and decision support system. Sustainability terms included drought tolerance, climate adaptation, conservation agriculture, and food security. The temporal scope covered publications from 2000 to February 2026; priority was given to work published after 2015 to ensure current relevance, while earlier seminal works were retained when they provided foundational theory.

### 2.3 Eligibility criteria

Candidate publications were evaluated against four inclusion criteria: (i) the study addressed one or more of the seven target cereal in relation to water use, irrigation management, or drought resilience; (ii) it reported quantitative data on water productivity, yield under water-limited conditions, or performance of water-saving technology; (iii) it appeared in a peer-reviewed journal, an established academic book series, or a technical report issued by a recognized international research organization; and (iv) it was in English. The following exclusion criteria were applied: (i) studies focusing exclusively on non-cereal crops; (ii) water-quality studies without relevance to crop water productivity; (iii) conference abstracts or editorials without original data; and (iv) duplicate records previously retrieved from alternative databases.

### 2.4 Study selection workflow and narrative-synthesis justification

**Figure 1:**
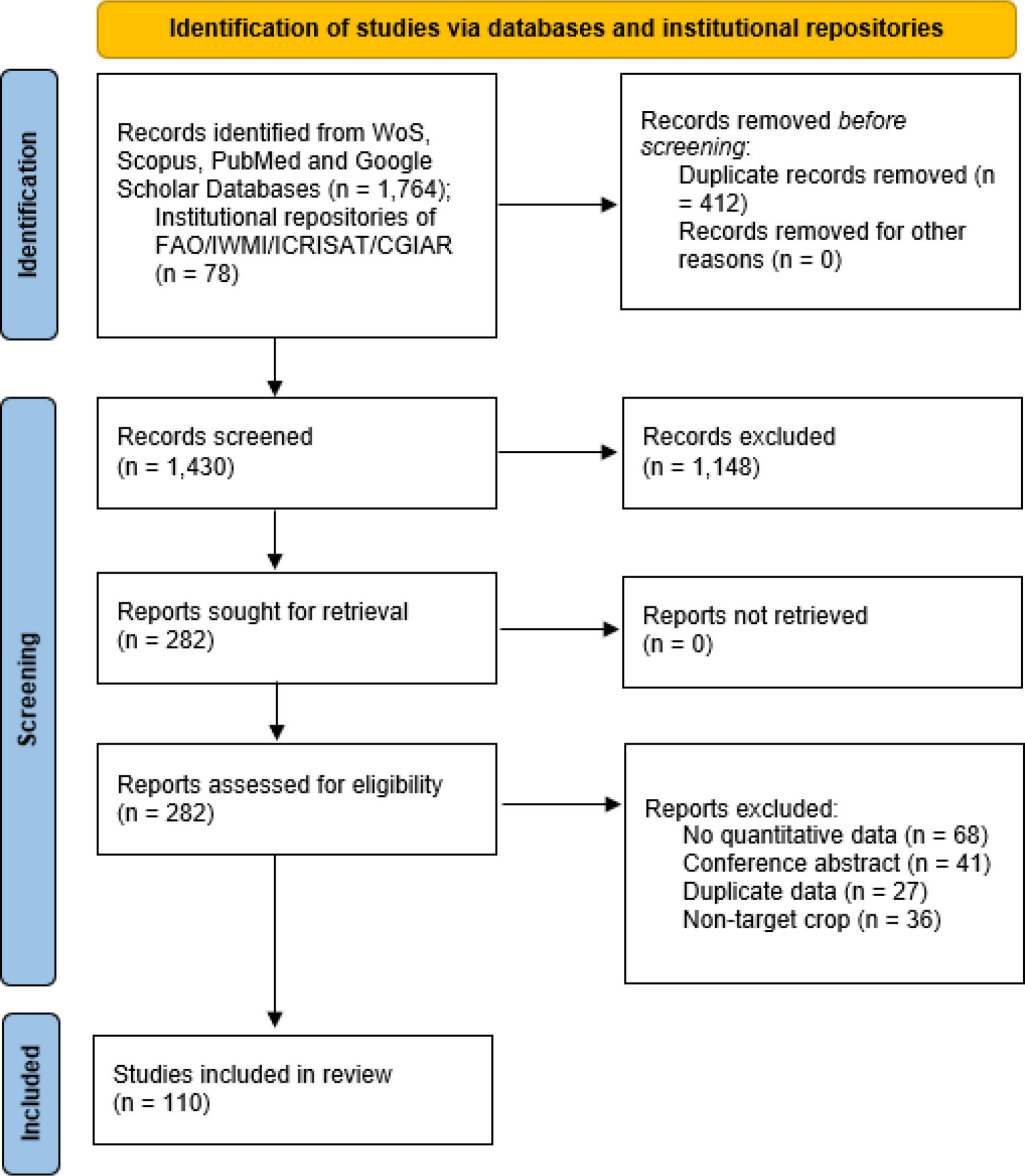
PRISMA-inspired study-selection flow.

The study-selection workflow is summarized in **Figure 1** (PRISMA-inspired flow). Records identified through database and repository searching (approximately 1,800 records) were deduplicated (approximately 1,430 after removal of duplicates). Title- and abstract-level screening excluded approximately 1,150 records (off-topic, non-cereal, non-English, or non-peer-reviewed). Full-text assessment was conducted on 282 records; 172 were excluded for various reasons. The final corpus comprises 110 references spanning semi-arid, sub-humid, Mediterranean, and irrigated lowland systems in Asia, sub-Saharan Africa, Latin America, and the Organization for Economic Co-operation and Development (OECD) member states.

A narrative-synthesis design was selected after considering the feasibility of a full systematic review with meta-analysis. The included evidence spans seven cereal species, six agroecological zones, and five intervention classes reported with incommensurate outcome metrics (grain yield, water productivity, irrigation water productivity, evapotranspiration, water footprint, economic return, time-to-adoption), which precludes valid statistical pooling across the whole scope (Greenhalgh et al., 2018). Where commensurate outcomes are reported within narrowly defined subdomains, published meta-analyses already exist (e.g., Carrijo et al., 2017, for AWD in rice; Qin et al., 2015, for mulching; Yu et al., 2020, for wheat deficit irrigation; Daryanto et al., 2016, for drought effects on maize and wheat; Cheng et al., 2021, for full, deficit, and partial root-zone irrigation). Their pooled effect sizes are cited throughout the present synthesis rather than re-pooled into a methodologically inferior meta-analysis.

Within each thematic domain, full-text evaluation followed a purposive strategy consistent with recommended practice for narrative reviews in the natural and applied sciences (Pautasso, 2013). Selection priority was given to: (i) meta-analyses and systematic reviews providing robust summary estimates; (ii) multi-year, multi-location field experiments with rigorous statistical designs and high external validity; (iii) simulation studies employing validated crop models (APSIM, AquaCrop, DSSAT) calibrated against independent field observations; and (iv) recent publications (2020–2026) documenting state-of-the-art developments in rapidly evolving fields such as IoT-based scheduling and AI-driven decision support. The reduced replicability of purposive selection relative to systematic protocols is acknowledged; the PRISMA-style flow and the explicit eligibility criteria are intended to improve transparency without claiming full systematic-review status.

### 2.5 Quality appraisal of included studies

Each included study was appraised qualitatively on four dimensions: (i) study design, ranked as meta-analysis/systematic review > multi-location field experiment > single-site field trial > crop-model simulation > narrative review; (ii) external validity, scored on number of sites, number of seasons, and agroecological representativeness; (iii) reporting transparency, assessed on availability of raw data, effect sizes, standard errors, and replicate numbers; and (iv) risk of bias, flagged where treatments lacked replication, controls were inadequate, or sponsor-affiliation conflicts of interest were plausible. The resulting quality tier (high, moderate, or exploratory) is reflected in the weight given to each finding in the narrative synthesis: quantitative ranges reported in the results sections and in **Table 1** are drawn predominantly from high-tier sources (meta-analyses and multi-location field trials), while findings from exploratory-tier sources are flagged as preliminary or proof-of-concept. The full tier classification is provided in **Supplementary Table 1**.

**Table 1:** Summary of deficit irrigation outcomes across major cereal crops.

Crop	Optimal deficit level	Water savings (%)	Yield change (%)	WP change (%)	Key findings and sources
Wheat	70–85% ETc	15–30	−5 to −16	+7 to +28^a^	Meta-analysis (41 studies): WP + 6.6%, yield −16.2% (Yu et al., 2020); 85% ETc and 70% ETc both optimal in Awash Basin; WUE 1.13–1.42 kg m^-^³ (Bayisa et al., 2024).
Maize	75% ETc (conv. furrow)	25–50	−5 to −23	+27 to +54^a^	Yield 101.2 vs 106.1 Qun ha^-^¹ at 75% ETc (−4.6%); 50% ETc: −22.8%; peak WP 8.0 kg m^-^³ (Ashine et al., 2024); regulated DI improves root: shoot ratio and WUE (Kang et al., 2000).
Rice (AWD)	Mild AWD (≥−20 kPa)	23–24	≈ 0 to −5	+24 to +31^a^	Meta-analysis 56 studies, 528 comparisons: mild AWD −23.4% water, non-significant yield loss (Carrijo et al., 2017); severe AWD −22.6% yield; 24% water savings in India (Sudhir-Yadav et al., 2011).
Sorghum	Mild–moderate deficit (50–75% ETc)	25–50	≈ 0 to +5	Increased^b^	HI exceeds full-irrigation value under mild–moderate stress; stay-green trait sustains post-anthesis C assimilation (Fereres and Soriano, 2007; Borrell et al., 2014).
Barley	Full ETc through anthesis; progressive drying during grain filling	Small to moderate	Small penalty under mild DI; larger under post-anthesis stress	Modest gain likely^b^	Early phenology permits drought escape; post-anthesis drought reduces tiller/spike number regardless of severity (Samarah, 2005; Fereres and Soriano, 2007).

^a^WP change calculated as irrigation water productivity: ((1 − yield-loss fraction)/(1 − water-savings fraction) − 1) × 100. ^b^Qualitative; WP increase follows from maintained or increased yield with reduced water input.

### 2.6 Data extraction and synthesis

From each included study, the following information was extracted where available: cereal crop(s) investigated, geographic location and climate zone, experimental design (field trial, modelling study, meta-analysis, or review), water-management intervention(s) evaluated, and primary quantitative outcomes reported (yield, water productivity, water footprint, or economic return). Where multiple studies reported comparable outcomes, ranges of reported values were synthesized in tabular form (**Tables 1**, **2**) to facilitate cross-study comparison. Given the methodological heterogeneity of the corpus, results are presented as narrative synthesis supported by tabular summaries rather than as pooled statistical effect sizes. The corpus distribution by thematic domain, crop, region, and study type is provided as **Supplementary Table 1** (updated in this revision to reflect the expanded coverage of sorghum, pearl millet, and sub-Saharan African smallholder systems).

**Table 2:** Critical synthesis of water-smart interventions: effects, trade-offs, and constraints.

Intervention class	Aggregated effect range	Principal trade-off/conflicting finding	Main implementation constraint	Strongest evidence source
Deficit irrigation (wheat, maize)	Water −15 to −50%; yield −5 to −23%; WP + 7 to +54%	WP gain traded for yield loss; benefit shrinks under higher-warming scenarios	Requires reliable rainfall forecasting and cultivar matching	Yu et al. (2020); Daryanto et al. (2016); Ren et al. (2022)
AWD (rice)	Water −23 to −24%; yield ≈ 0 (mild) to −23 (severe)	Severe drying causes disproportionate yield loss; soil pH ≥ 7 or SOC < 1% amplifies risk	Requires field-level water-depth monitoring and farmer training	Carrijo et al. (2017); Lampayan et al. (2015)
Mulching (plastic)	Maize yield +30–50%; WP + 25–45%	Persistent microplastic residue; uncertain field-scale biodegradability of alternatives	End-of-life collection and disposal logistics	Bu et al. (2013); Sintim and Flury (2017)
Mulching (straw)	Maize yield +22%, wheat +13% (pooled)	Competes with livestock-feed use in smallholder systems	Requires residue-retaining harvest and sowing equipment	Qin et al. (2015); Chai et al. (2022)
Conservation agriculture	Available soil water +~10%; wheat yields +4–6%; −1 irrigation season^-^¹	Short-term weed and N-immobilization penalties; residue burning is a confounder	Residue-handling seed drills and extension support	Erenstein and Laxmi (2008); Palm et al. (2014); Blanco-Canqui and Ruis (2018)
Drought-resilient cultivars	Yield within 5–8% of well-watered under terminal drought (stay-green sorghum)	Polygenic WP limits MAS efficacy; IP/seed-system barriers	Seed-system reach; participatory variety selection	Borrell et al. (2014); Singh et al. (2025); Sehgal et al. (2023)
IoT sensor scheduling	Meaningful but heterogeneous water savings vs calendar-based; cereal-specific evidence base thin	Single-site cereal trials dominate; savings not yet robustly multi-site	Upfront cost, rural connectivity, technical capacity	Abdelmoneim et al. (2025); Navarro-Hellín et al. (2015); Bwambale et al. (2022)
AI/DSS-driven scheduling	Magnitude strongly dependent on comparator and configuration	Most evidence extrapolated from horticulture; cereal-specific RCTs scarce	Data-governance regime; open-source availability	Sharma et al. (2021); Ali et al. (2023); Liakos et al. (2018)
Integrated G×A×D packages	Package-specific; Indo-Gangetic ZT+AWD: ~1 irrigation saved + 23% water saving in rice	Adoption depends on institutional alignment, not only technical fit	Water-user associations; extraction caps; cooperative governance	This review (**Figure 2**; Section 8.1)

## 3 Water use dynamics in global cereal production systems

### 3.1 Crop-specific water requirements and water productivity

The water requirements of cereal crops vary substantially by species, growth stage, and production environment. Rice is by far the most water-demanding cereal, with conventional flooded paddy systems requiring 1200–2000 mm of water per cropping season, compared with 450–650 mm for wheat and 500–800 mm for maize under typical irrigated conditions (Bouman et al., 2005; Rattalino Edreira et al., 2018). Paddy rice maintains standing water to suppress weeds and maintain anaerobic soil conditions (Bouman and Tuong, 2001), whereas C4 cereals such as maize and sorghum have inherently higher transpiration efficiency owing to their carbon-concentrating mechanisms (Sage, 2004).

Water productivity (WP), defined as grain yield per unit of evapotranspiration (ET), provides a more meaningful cross-environment metric than water use alone. Among major cereals, maize generally shows the highest WP (1.3–2.7 kg m^-^³), particularly under optimized deficit irrigation (Chahal et al., 2007; Kingra et al., 2019), followed by sorghum (1.24–1.34 kg m^-^³) (Maman et al., 2003; Araya et al., 2017), and wheat (0.22–1.60 kg m^-^³) (Usman et al., 2014; Foley et al., 2020). Pearl millet tends to be lower (0.51–1.04 kg m^-^³) (Maman et al., 2003), while rice generally records the lowest values, ranging from 0.65 to 0.79 kg m^-^³, primarily due to the high-water consumption associated with traditional flooded paddy systems (Chahal et al., 2007; Usman et al., 2014; Foley et al., 2020).

### 3.2 Drivers of inter-crop and inter-regional variability in water productivity

Descriptive comparisons of WP are most informative when supported by an analytical account of the underlying drivers. Four principal drivers can be identified from the evidence base:

- Photosynthetic pathway: The C4 pathway of maize, sorghum, and millets sustains CO_2_ assimilation at lower stomatal conductance than the C3 pathway of wheat, barley, and rice, and accounts for much of the systematic WP advantage of C4 cereals in warm, high-radiation environments (Sage, 2004). This mechanism explains why maize WP can reach 2.7 kg m^-^³ even in semi-arid settings where wheat rarely exceeds 1.6 kg m^-^³.
- Canopy and root architecture: Short-season cereals with determinate phenology and deep root systems (e.g., barley, early-flowering wheat) achieve higher WP under terminal drought because their yield formation window avoids the period of highest atmospheric evaporative demand. The stay-green trait in sorghum (Borrell et al., 2014) extends this logic by sustaining post-anthesis canopy photosynthesis under progressive soil drying.
- Climatic vapor-pressure deficit (VPD): The three-decade meta-analysis of Foley et al. (2020) across 148 irrigated sites in 31 countries shows that wheat WP declines systematically as mean growing-season VPD increases; this explains the large latitudinal WP gradient between temperate OECD systems and sub-tropical monsoon systems that cannot be attributed to genotype alone.
- Irrigation technology and management: Cross-country comparisons show that wheat WP in high-efficiency systems of the USA and China can exceed values in India by a factor of two to five at comparable VPD (Foley et al., 2020), reflecting the combined effect of field levelling, conveyance losses, fertigation management, and cultivar choice rather than inherent climatic limits. This finding has direct policy implications: even a 10% improvement in Indian wheat WP alone would yield water savings of the order of several thousand billion liters per year.

Taken together, these drivers suggest that investment in WP improvement should be targeted differentially. Genotype-based gains (drivers 1 and 2) are most tractable in breeding pipelines with a multi-trait focus (Chenu et al., 2018; Sellami et al., 2024); VPD-related gains (driver 3) require sowing-date adjustment and heat-tolerant germplasm (Asseng et al., 2014; Minoli et al., 2022); management-related gains (driver 4) require irrigation-system upgrading, extension services, and appropriate water-governance instruments (Grafton et al., 2018).

### 3.3 The water footprint challenge

The water footprint framework provides a comprehensive accounting of the total freshwater consumed in cereal production, distinguishing green (rainfall), blue (irrigation), and grey (pollution dilution) water (Hoekstra et al., 2011). Global resolved assessments quantify the water footprint of wheat at 1,827 m³ t^-^¹, followed by rice at approximately 1,673 m³ t^-^¹ and maize at 1,222 m³ t^-^¹—the latter representing approximately 27% less water per unit of production than rice and approximately 33% less than wheat (Mekonnen and Hoekstra, 2011).

India illustrates the scale of the challenge: it carries the highest national freshwater demand globally, 91% of which is allocated to agriculture, with cereals accounting for more than 50% of the dietary water footprint (Davis et al., 2018). Historical analyses indicate that the expansion of wheat cultivation in the dry (rabi) season has been the principal driver of increasing consumptive irrigation-water demand, while rice remains the least water-efficient cereal per unit of iron, zinc, and dietary fiber produced (Davis et al., 2018).

Replacing rice with alternative cereals such as maize, sorghum, and millets in suitable agroecological zones could simultaneously reduce freshwater use and enhance nutrient provision without expanding cropland (Davis et al., 2018). Current yields of millets and sorghum remain insufficient to fully offset a reduction in rice output, so achieving a large-scale transition requires sustained investment in the productivity of drought-resilient cereals alongside structural shifts in consumer preferences and public procurement (Harris et al., 2019).

## 4 Deficit irrigation strategies for cereal crops

### 4.1 Principles and physiological basis

Deficit irrigation (DI) is a deliberate water management strategy in which applied water is maintained below full crop evapotranspiration (ETc) during part or all of the growing season to maximize water productivity rather than absolute yield (Fereres and Soriano, 2007). The agrophysiological rationale is grounded in the nonlinear relationship between cumulative seasonal ET and grain yield: as water supply declines from full ETc, yield initially falls more slowly than water savings because the crop conserves its harvest index (HI) through reduced vegetative biomass, earlier canopy senescence, and enhanced remobilization of pre-anthesis stem reserves to the grain (Steduto et al., 2009).

These compensatory responses are bounded by crop- and cultivar-specific thresholds. In bread wheat, the yield–ET relationship remains approximately linear until about 60% of fully irrigated biomass, below which HI declines sharply as assimilate supply fails to meet grain demand (Fereres and Soriano, 2007). A global meta-analysis of 41 studies confirmed that deficit irrigation in wheat improved WP by a pooled mean of 6.6% but reduced yield by 16.2%, with the optimal balance achieved when irrigation was reduced during the vegetative rather than the reproductive phase (Yu et al., 2020). Sorghum, by contrast, exhibits a distinctive adaptive advantage: under mild-to-moderate water deficit (50–75% ETc), HI can exceed values obtained under full irrigation because the stay-green trait prolongs post-anthesis photosynthesis and sustains assimilate translocation (Fereres and Soriano, 2007; Borrell et al., 2014).

The sensitivity to water deficit is strongly phenology-dependent. In rice, moderate wetting and drying during the vegetative phase reduces excessive tillering and improves the canopy structure and HI without lowering the grain yield (Yang and Zhang, 2010). The meta-analysis of Daryanto et al. (2016) reported that maize is substantially more sensitive to water deficit during the reproductive phase (39.3% yield reduction at ~40% water reduction) than wheat (20.6%), and that maize is particularly vulnerable during flowering, whereas wheat exhibits more uniform sensitivity across vegetative and reproductive stages. Post-anthesis water deficits are generally the most damaging interval for all cereals because they shorten grain filling duration and reduce final grain weight (Fereres and Soriano, 2007).

In lowland irrigated rice, DI takes the specific form of alternate wetting and drying (AWD): paddy fields dry to a predetermined soil water potential before re-flooding, rather than maintaining a continuous standing water (Lampayan et al., 2015). The critical management parameter is the severity of drying between re-flooding events. In the meta-analysis of 56 studies with 528 paired comparisons by Carrijo et al. (2017), mild AWD (soil water potential ≥ −20 kPa, or field water level not dropping below 15 cm from the soil surface) reduced water inputs by 23.4% with no significant yield penalty in most circumstances, whereas severe AWD incurred yield losses of 22.6%, greatest in soils with pH ≥ 7 or organic carbon below 1%. AWD is now recommended by the International Rice Research Institute (IRRI) and has been adopted across irrigated rice systems in Bangladesh, the Philippines, and Vietnam (Lampayan et al., 2015). Across cereal and horticultural crops more broadly, DI consistently increases WP relative to full irrigation, although the magnitude is strongly modulated by crop, soil texture, climate aridity, and the phenological window targeted (Geerts and Raes, 2009; Cheng et al., 2021).

### 4.2 Critical evaluation: trade-offs, risk envelopes, and scaling limits

The aggregate evidence on DI shows a clear yield–water trade-off that varies markedly by crop and environment (**Table 2**). Wheat typically loses 5–16% of yield for water savings of 15–30% (Yu et al., 2020; Bayisa et al., 2024); maize can lose 5–23% of yield for water savings of 25–50% (Ashine et al., 2024); rice under mild AWD achieves ~23% water savings without statistically significant yield penalty but tips into a ~23% yield loss under severe drying (Carrijo et al., 2017); sorghum under mild deficit often maintains or marginally increases yield because of the stay-green HI response (Fereres and Soriano, 2007; Borrell et al., 2014). These asymmetries reflect differences in the duration of the yield-formation window, the flexibility of the harvest index, and the depth and plasticity of root-water capture.

The climatic-risk envelope in which DI becomes counterproductive is definable. DI is least robust, and most likely to deliver yield losses disproportionate to water savings, in arid climates with high inter-annual rainfall variability, in soils with low plant-available water-holding capacity, and in seasons where post-anthesis rainfall is highly uncertain. Under such conditions the assumption that crops can compensate for reduced irrigation through enhanced root-water capture or remobilization often fails (Fereres and Soriano, 2007). The projected trajectory of DI performance under climate change compounds this concern: the crop-model analysis of Ren et al. (2022) using 21 CMIP6 general-circulation models shows that the yield and WP benefit of DI in the North China Plain diminishes under SSP245 and SSP585, principally because increased growing-season rainfall reduces the relative advantage of strategic irrigation timing. This indicates that DI, effective under present climate variability, will need to be coupled with heat-tolerant germplasm and adjusted sowing windows to remain a viable long-term adaptation (Minoli et al., 2022).

Practically, DI is most robust when deployed as a component of an integrated package rather than in isolation. Evidence from the semi-arid Loess Plateau shows that ridge-and-furrow rainwater-harvesting structures can increase maize yield by 21%, WP by 17%, and precipitation-use efficiency by 22% when combined with DI at tasseling–silking (Ren et al., 2017). Straw strip-mulching combined with DI has been shown to deliver winter-wheat yields comparable to those of full plastic-film mulching (Chai et al., 2022). In Ethiopia, reducing applied water to 85% ETc cut wheat yield by only ~5%, while 55% and 40% ETc reduced yield by 32% and 51%, respectively (Bayisa et al., 2024), underscoring that the benefit of DI is bounded and crop-specifically calibrated. The implication for practitioners is that DI should be adopted where supplemental tools (micro-catchments, mulching, drought-tolerant cultivars) can buffer the downside risk, and avoided where such tools are not available.

### 4.3 Crop-specific deficit irrigation optimization

Recent experimental and modelling evidence has refined DI recommendations for specific cereal systems. In the North China Plain, where intensive wheat-maize double-cropping has placed severe pressure on groundwater, simulation analyses with APSIM driven by 21 CMIP6 climate models show that moderate DI applied at a trigger threshold of 40% of plant-available water capacity during the sowing-to-flowering period, combined with nitrogen fertilization at 150 kg N ha^-^¹, represents the optimum scheduling strategy that balances yield stability and water productivity under both current and projected climate conditions (SSP245 and SSP585) (Ren et al., 2022). China’s irrigation water productivity has risen from 0.2 kg m^-^³ in the 1950s to 1.58 kg m^-^³ by 2013, and regulated deficit irrigation— particularly alternate partial root-zone irrigation — has been central to this progress (Kang et al., 2017).

For maize, Ren et al. (2017) demonstrated on the semi-arid Loess Plateau of China that DI at the tasseling-to-silking stage under ridge-and-furrow rainfall harvesting systems raises grain yield by 21%, WP by 17%, and precipitation-use efficiency by 22% relative to conventional level-bed sowing. For wheat in the semi-arid Awash Basin of Ethiopia, reducing applied water to 85% ETc cut yield by only 5% relative to full irrigation, whereas 55% and 40% ETc caused 32% and 51% yield losses, respectively (Bayisa et al., 2024), identifying mild DI as a productive option in that setting.

For barley, which is frequently grown as a terminal-drought crop in Mediterranean and highland environments, early phenology and rapid grain filling provide a degree of drought escape, DI strategies that maintain water supply through anthesis while permitting progressive drying during grain filling can sustain acceptable yield (Fereres and Soriano, 2007). However, greenhouse studies show that post-anthesis drought stress reduces yield regardless of severity by shortening the grain-filling period and reducing tiller number, spike number, and individual grain weight (Samarah, 2005). Sorghum combines DI with the stay-green trait to maintain or even increase yield under 50–75% ETc regimes, making it the cereal with the widest adaptive margin for DI-based water savings (Fereres and Soriano, 2007; Borrell et al., 2014).

DI is most effective when paired with stay-green sorghum germplasm or deep-rooting wheat cultivars (Section 6), and with soil-moisture conservation practices (Section 5) that buffer the downside risk during dry spells. The integration of DI with IoT-based sensor scheduling (Section 7) can in principle shift the operator from calendar-based toward physiologically-based water application, but the cereal-specific field evidence for this integration is still at the proof-of-concept stage (Section 7.3).

## 5 Soil moisture conservation strategies

### 5.1 Mulching technologies

Across the 74-study meta-analysis of Qin et al. (2015), soil mulching significantly increased maize and wheat yields and water productivity, with straw mulching raising cereal yields by a pooled mean of 13–22% and plastic-film mulching by a larger margin in cold or semi-arid conditions; the effect decreased as baseline water input approached full ET. Mulching is therefore among the most widely studied and consistently effective agronomic interventions for conserving soil moisture in cereal production. Its main physical mechanism is suppression of direct soil evaporation (stage-I evaporative loss), which can account for 30–60% of total seasonal ET in cereals sown at wide row spacing under semi-arid conditions (Allen et al., 1998; Kader et al., 2017). Mulches interpose a barrier between soil and atmosphere, attenuate the vapor pressure gradient at the soil–air interface, reduce diurnal soil temperature oscillations, and impede wind-driven moisture removal (Kader et al., 2017). Secondary benefits include improved rainfall infiltration through protection against raindrop impact and crust formation, suppression of weed emergence and competitive transpiration, and—for organic mulches— progressive improvement of soil organic carbon, aggregate stability, and biological activity (Qin et al., 2015; Kader et al., 2017; Mulumba and Lal, 2008).

The efficacy of mulching varies substantially with material type, application rate, climate, and soil texture. Synthetic mulches, particularly black polyethylene film (0.008–0.01 mm thickness), deliver the greatest short-term moisture conservation because they are essentially impermeable to water vapor. In rainfed maize systems on the semi-arid Loess Plateau, plastic-film mulching raised maize yield and WP by 30–50% relative to unmulched controls (Bu et al., 2013). However, plastic film mulch has well-documented environmental liabilities, treated in the next paragraph.

#### 5.1.1 Environmental risks of plastic mulch

Plastic-film mulching generates persistent soil contamination: residual fragments accumulate in the plough layer over successive seasons and release micro-plastics into the terrestrial environment (Sintim and Flury, 2017). Three concerns require explicit recognition. First, so-called biodegradable plastic mulch films have uncertain field-scale behavior and it is not established that they fully degrade under field conditions in realistic time horizons (Sintim and Flury, 2017). Second, some films contain additives (phthalate plasticizers, UV stabilizers) with documented endocrine-disrupting potential that may leach into the root zone. Third, end-of-life film collection and disposal carry substantial logistical and economic costs that are rarely internalized in productivity comparisons with organic alternatives. The short-term WP gains of plastic mulch must therefore be weighed against these long-term externalities. Straw strip-mulching (Chai et al., 2022) can achieve comparable hydrothermal outcomes — +27% grain yield, +25 mm soil-water storage in the 0–200 cm profile, net income 30% above unmulched controls — without these environmental costs and should, where crop residues are available, be preferred on sustainability grounds.

Organic mulches—predominantly cereal straw, but also rice husks, maize stover, and composted manure—provide a more sustainable alternative. The Qin et al. (2015) meta-analysis found that straw mulching effects are larger at higher temperatures and in low-input systems, confirming its value for dryland cereal production. Upon decomposition, straw residues improve aggregate stability, increase available water capacity by 18–35%, and enhance total porosity by 35–46%, creating a positive feedback loop that progressively raises the soil’s intrinsic moisture retention (Mulumba and Lal, 2008).

#### 5.1.2 Context-specific effectiveness

The effectiveness of mulching is not universal. A multi-country coordinated project across arid and semi-arid regions (seasonal rainfall generally <300 mm) found that mulching effects on crop yield were inconsistent: while maize yields increased with mulching in China, similar effects were not observed in wheat, and other soil-water-conservation practices including ridging and zero tillage did not show significant yield effects under very low soil-moisture regimes (IAEA, 2005). The synthesis concluded that when vegetative-stage soil moisture is extremely low because of low or erratic rainfall, the expected benefits of moisture conservation methods cannot be achieved (IAEA, 2005). Three decision criteria are therefore proposed for site-specific mulching recommendations: (i) within-season rainfall distribution (mulching benefits are greatest where rainfall is moderate and variable, rather than absent); (ii) soil hydraulic properties (benefits are larger in coarser-textured soils with low intrinsic moisture retention); and (iii) the availability and opportunity cost of mulching material (in rainfed smallholder systems the straw may be needed for livestock feed, raising a competing claim on residues).

### 5.2 Conservation tillage and residue management

While mulching addresses the evaporative component of the field water balance, conservation agriculture (CA) targets the infiltration and storage components through a systems-level approach consistent with the broader rainfed water-productivity framework developed by Rockström and Barron (2007). CA is defined by three interlinked principles: (i) minimum mechanical soil disturbance (zero-tillage or reduced tillage), (ii) permanent organic soil cover through crop residues or cover crops, and (iii) diversified crop rotations (Kassam et al., 2018). By eliminating or minimizing tillage, CA preserves the continuity of soil macropores, including biopores created by earthworms and decayed root channels, that serve as preferential pathways for rainfall infiltration (Blanco-Canqui and Ruis, 2018). Undisturbed surface residues protect the soil from raindrop impact, reduce crust formation, and slow surface runoff, increasing the proportion of rainfall that enters the soil profile. Over multiple seasons, organic matter accumulation in the surface horizon improves aggregate stability and raises available soil water by approximately 10% relative to conventional tillage, with gains concentrated in the upper soil layers (Palm et al., 2014; Blanco-Canqui and Ruis, 2018).

The quantitative evidence for CA-induced water savings is best documented in the Indo-Gangetic Plains, where the rice–wheat rotation occupies approximately 13.5 million hectares across India, Pakistan, Nepal, and Bangladesh. Conventional practice involves intensive tillage for wheat-bed preparation following puddled rice, which degrades soil and threatens system sustainability (Erenstein and Laxmi, 2008). Zero-till wheat, drilled directly into unploughed fields, has consistently saved roughly one irrigation per season across on-farm evaluations in the western Indo-Gangetic Plains, owing to preserved soil structure retaining residual moisture from the preceding rice crop and the earlier sowing window reducing bare-soil evaporation (Erenstein and Laxmi, 2008). Combined with 4–6% yield gain, this translates into a meaningful improvement in crop water productivity. Nevertheless, prevailing zero-till seed drills handle crop residues poorly, and where rice is combine-harvested, straw burning has remained common—forgoing the additional evaporation-suppression benefit that surface residue retention would provide (Erenstein and Laxmi, 2008).

Cover cropping, the third pillar of CA, can further enhance subsequent cereal water productivity by improving the soil physical environment between cash crop seasons. Deep-rooted species such as forage radish (*Raphanus sativus* L.) and rapeseed (*Brassica napus* L.) penetrate compacted subsoil more effectively than fibrous-rooted species such as rye, creating bio-channels that persist after root decay and improve water and air flow into horizons that are otherwise difficult for cereal roots to exploit (Blanco-Canqui et al., 2015). Upon termination, cover crop residues form a surface mulch that intercepts rainfall, reduces runoff and sediment loss, and suppresses evaporation, while decomposing root biomass contributes to soil organic carbon and aggregate stability (Dabney et al., 2001; Blanco-Canqui et al., 2015).

#### 5.2.1 Critical trade-off

For cereal systems, improved subsoil macroporosity is particularly valuable because it allows maize and wheat roots to access deeper stored moisture during grain filling—the growth stage most sensitive to water stress (Blanco-Canqui et al., 2015). However, these benefits entail a well-documented trade-off: actively transpiring cover crops consume soil water during their growth period, and in water-limited environments, the residual soil moisture deficit at cover-crop termination can reduce yields for the subsequent cash crop (Blanco-Canqui et al., 2015). Managing this trade-off requires attention to termination timing—cover crops should be terminated early enough to allow soil water recharge before cash crop establishment—and the choice of species and mixtures suited to local water availability (Blanco-Canqui et al., 2015).

#### 5.2.2 Integration with other levers

Soil moisture conservation (SMC) practices complement DI (Section 4) by reducing the downside yield risk of water restriction, and complement drought-resilient varieties (Section 6) by increasing the soil-moisture reservoir from which deeper-rooted cultivars can draw. SMC also provides the biophysical substrate on which precision-irrigation systems (Section 7) operate: sensor networks deployed in CA fields generally report lower spatial variability and greater signal stability than those deployed in conventionally tilled fields.

## 6 Deployment of drought-resilient cereal varieties

### 6.1 Conventional breeding and field-level evidence

Development of drought-tolerant cereal varieties through conventional breeding directly underpins water-productivity improvement, because genetic gains in yield per unit water consumed complement agronomic gains from improved water management. Passioura’s foundational framework decomposes cereal grain yield under water limitation into three multiplicative components: total water use, transpiration efficiency, and harvest index (Passioura, 1977). Breeding programs have targeted all three, selecting for deeper and more vigorous root systems that access subsoil moisture and increase total water capture; early flowering and maturity that match crop demand to the period of water availability; higher transpiration efficiency linked to carbon-isotope discrimination; and enhanced assimilate remobilization from stem reserves to grain under terminal drought (Richards, 2006). In Australian dryland wheat, Turner (2004) showed that at least half of the historical increase in rainfall-use efficiency was attributable to improved agronomic management—minimum tillage, fertilizer use, and weed control—while the remainder reflected genetic improvement, underscoring that varietal development and on-farm practices are synergistic rather than competing pathways to higher WP.

#### 6.1.1 Multi-location field evidence for stay-green sorghum

The clearest demonstration of translational impact in drought-resilient cereal breeding comes from the stay-green sorghum program. Multi-location trials across Australia, India, and West Africa have shown that stay-green hybrids maintain grain yield within 5–8% of well-watered controls under terminal drought, an outcome mechanistically linked to delayed post-flowering leaf senescence, sustained canopy photosynthesis during grain filling, and deeper water uptake from the subsoil (Borrell et al., 2014). These results exemplify how a single well-characterized trait, deployed in locally adapted backgrounds, can deliver real-world yield stability in water-limited environments.

Sorghum and pearl millet are naturally well-suited to water-limited environments because their C4 photosynthetic pathway confers higher transpiration efficiency than the C3 pathway of wheat and rice. Sorghum’s drought adaptation includes an extensive root system for deep soil water extraction, a waxy cuticle that reduces non-stomatal water loss, and osmotic adjustment that maintains cell turgor under declining water potential (Rosenow et al., 1983). Pearl millet (*Pennisetum glaucum* [L.] R. Br.) is similarly adapted to the driest cereal-growing environments of Africa and Asia, where it produces viable yields under seasonal rainfall as low as 200–300 mm (Rai et al., 2008). Both crops offer nutritional advantages over wheat or rice, with higher iron and zinc concentrations, reinforcing the food security rationale for their inclusion in water-smart cropping systems (Rai et al., 2008). Finger millet (*Eleusine coracana* [L.] Gaertn.), although underrepresented in the global research literature, plays a similarly important role in high-altitude and semi-arid areas of East Africa and India; the absence of a strong field-trial evidence base for finger millet is one of the clearest priority gaps identified by this review.

#### 6.1.2 Indigenous dryland cereals and seed-system equity in sub-Saharan Africa

The deployment of drought-resilient sorghum and millet varieties in sub-Saharan African smallholder systems is constrained less by the availability of improved germplasm than by the reach of formal seed systems. Informal farmer-to-farmer seed exchange accounts for the majority of sorghum and millet seed used in many West and East African countries (Almekinders and Louwaars, 2002; McGuire and Sperling, 2016), which limits the speed at which breeder-developed cultivars reach end-users. Evidence from participatory variety-selection programs demonstrates that farmer-preferred varieties differ systematically from breeder-preferred varieties on traits such as threshability, grain color, and post-harvest storability, and that gender-differentiated preferences are particularly pronounced in African dryland cereals, with men and women often prioritizing different trait combinations (Weltzien et al., 2019). These seed-system and social-preference dynamics mean that the translation of genetic gains into aggregate water-smart outcomes depends critically on the seed-delivery infrastructure and on participatory varietal selection, not only on the breeding pipeline itself.

### 6.2 Molecular breeding, genomic tools, and their field-translation limits

Advances in molecular biology have expanded the toolkit for accelerating genetic gains. Marker-assisted selection (MAS) enables breeders to track specific genomic regions (quantitative trait loci, or QTLs) associated with drought-adaptive traits and to introgress favorable alleles into elite cultivar backgrounds without the lengthy phenotypic cycles of conventional selection (Singh et al., 2025). Marker-assisted recurrent selection (MARS) pyramids multiple QTLs in a single population through iterative genotypic selection (Singh et al., 2025). High-density genotyping platforms such as the wheat 660K SNP array have enhanced the resolution of QTL mapping and GWAS for drought-adaptive traits (Sehgal et al., 2023). For highly polygenic traits such as water productivity, genomic selection (GS)— using genome-wide marker data to predict breeding values without prior QTL knowledge —is emerging as a complementary approach, although its cereal-specific validation for drought tolerance remains at an earlier stage than MAS (Sehgal et al., 2023).

Gene-editing technologies, particularly CRISPR-Cas9, offer the potential to modify genes involved in crop water balance precisely, and in some delivery systems can generate edited plants without foreign DNA in the final product (Zaidi et al., 2020). Targets in cereal drought-tolerance research include genes regulating stomatal development and aperture control, root architecture, osmolyte-associated pathways, and transcription-factor networks such as DREB/CBF (Zaidi et al., 2020; Singh et al., 2025). Transgenic studies have provided proof-of-concept evidence: expression of the barley HVA1 gene, encoding a group-3 late-embryogenesis-abundant protein, enhanced tolerance to water deficit and salinity in rice under controlled conditions (Xu et al., 1996).

#### 6.2.1 Critical limitations of molecular tools

Four limitations require explicit acknowledgement. First, a persistent lab-to-field translation gap exists: several transgenic and genome-edited drought-tolerance events that performed well under controlled conditions have not robustly translated into farmer-field yield gains, because laboratory stress regimes rarely replicate the multi-stress, multi-season reality of field environments (Luo, 2010). Second, the polygenic architecture of water productivity limits the effectiveness of MAS for yield-under-drought per se, favoring genomic selection — for which cereal-specific, high-quality training populations remain under-resourced, particularly for orphan cereals. Third, the regulatory environment for genetically modified and genome-edited crops is highly heterogeneous across jurisdictions, and the resulting fragmentation of global deployment pathways increases both the cost and the time-to-market for molecular improvements (Zaidi et al., 2020). Fourth, intellectual-property regimes and seed-system constraints can prevent smallholders from accessing improved germplasm, particularly for crops (sorghum, millets) that are of low commercial interest to multinational breeders. These four factors collectively mean that the contribution of molecular tools to cereal water-smart outcomes over the coming decade is likely to be incremental rather than transformational, and complementary to — not a substitute for — conventional breeding and agronomic improvement.

#### 6.2.2 Integration with other levers

Drought-resilient varieties deliver the greatest WP gains when matched to the agronomic and digital environment in which they are deployed. Stay-green sorghum hybrids perform best under mild-to-moderate deficit-irrigation regimes (Section 4) that exploit their stay-green HI response. Deep-rooted wheat cultivars deliver greater additional benefit in conservation-agriculture fields (Section 5) than in ploughed fields, because the undisturbed macropores amplify their root access. Sensor-based irrigation scheduling (Section 7) can in principle be calibrated to the physiological thresholds of drought-tolerant cultivars, but cultivar-specific parameterization of commercial decision-support systems is not yet widespread.

## 7 Precision agriculture and digital irrigation technologies

Digital irrigation technologies are frequently promoted with considerable enthusiasm, but the published evidence base for their cereal-specific field performance remains thinner than the pace of commercial marketing would suggest. This section accordingly reports only those applications that have been validated in cereal field trials, flags where published claims are extrapolated from horticulture or other high-value crops, and concludes with a dedicated subsection (7.4) on economic feasibility, adoption barriers, and equity considerations.

### 7.1 Remote sensing for crop-water-status monitoring

Remote sensing can enhance cereal WP by enabling spatially explicit monitoring of crop water status, soil-moisture variability, and vegetation condition at scales relevant to targeted management (Weiss et al., 2020). Satellite platforms such as Sentinel-2 and Landsat-8 provide freely available multispectral imagery from which vegetation indices, including NDVI and other water stress-related metrics, can be derived at field and sub-field scales to identify zones where water limitation constrains transpiration and yield (Weiss et al., 2020). The Optical Trapezoid Model (OPTRAM), when used with Sentinel-2 optical and shortwave-infrared data, has shown promise for high-resolution surface soil moisture estimation with modest ground-calibration requirements, although local calibration remains important to ensure accuracy under different soil and climate conditions (Hassanpour et al., 2020; Ma et al., 2022).

Unmanned aerial vehicles (UAVs) equipped with multispectral, thermal, and hyperspectral sensors complement satellite observations by providing on-demand ultra-high-resolution data that captures within-field variability in crop water stress (Maes and Steppe, 2019). UAV-based thermal imagery can detect canopy-temperature rises associated with stomatal closure and reduced transpiration before visible wilting develops (Gago et al., 2015). Reflectance indices derived from UAV multispectral data, including NDVI, TCARI/OSAVI, and PRI, show useful relationships with water-stress indicators such as leaf water potential and stomatal conductance, although performance depends on crop type and environmental conditions (Gago et al., 2015). Much of the published UAV-irrigation-management literature concerns orchards, vineyards, and vegetables; cereal-specific multi-site field validation is less abundant and should be an explicit research priority.

Combining satellite and UAV observations with geographic information systems supports variable-rate-irrigation maps (Hedley and Yule, 2009). This approach is becoming more common for cereal systems with substantial within-field variability in soil and water conditions, but its commercial deployment in smallholder cereal systems of the global South remains limited by the cost and operational complexity of the associated machinery.

### 7.2 IoT-based soil moisture monitoring and smart irrigation

Internet of Things (IoT) technologies enable continuous, near-real-time monitoring of soil moisture, weather, and field status through networks of wireless sensors deployed at field scale (Bwambale et al., 2022). Contemporary IoT-based irrigation systems integrate soil-moisture sensors, on-site weather measurements, and decision-support models to estimate site-specific irrigation requirements and better align water application with crop demand (Bwambale et al., 2022). Data transmission typically relies on low-power, long-range communication technologies such as LoRa, ZigBee, and cellular networks, cloud-based platforms increasingly incorporate analytics and, in some cases, machine-learning tools to generate irrigation recommendations or automate valve control (Li et al., 2020; Abdelmoneim et al., 2025).

The systematic review of Abdelmoneim et al. (2025) synthesizes IoT-irrigation publications from the late 2010s to 2024 and reports that cereal-focused studies generally show meaningful irrigation-water savings relative to calendar-based scheduling, with yield outcomes ranging from small reductions to modest gains. Reported savings vary substantially across studies and agroecological settings, reflecting differences in baseline scheduling practice, sensor type, crop, and season. The authors note that most of the reviewed cereal studies are single-site, single-season demonstrations, and that multi-site cereal-specific randomized or paired-field trials remain rare. This field-scale picture is consistent with earlier global-simulation work showing that a shift from traditional surface irrigation to improved scheduling and delivery could save large volumes of water at the system scale (Jägermeyr et al., 2015). Earlier wireless-sensor-network work established the technical feasibility of real-time sensor-driven irrigation scheduling at field scale (Navarro-Hellín et al., 2015), and broader reviews of IoT in agro-industrial systems have documented the growing integration of sensor networks with decision-support tools (Talavera et al., 2017). Nevertheless, quantitative evidence of IoT-driven gains in WP is still more limited for cereal systems than for horticultural and other high-value crops (Bwambale et al., 2022). Important barriers to wider adoption — upfront costs, limited rural connectivity, technical-capacity and maintenance demands, data-governance concerns — are treated explicitly in Section 7.4 (Elijah et al., 2018).

### 7.3 Artificial intelligence and decision-support systems

Artificial intelligence (AI), particularly machine learning and deep learning, is increasingly applied in precision irrigation to align water supply with crop demand (Ali et al., 2023). Machine-learning models trained on combinations of historical weather data, soil properties, crop information, and real-time sensor inputs have been applied to predict soil-moisture dynamics and estimate irrigation requirements, supporting more proactive scheduling (Liakos et al., 2018; Sharma et al., 2021). Reviews indicate that such intelligent irrigation systems can reduce water use relative to conventional fixed or calendar-based scheduling; however, the magnitude of savings varies with crop type, climate, system configuration, and the baseline comparator (Sharma et al., 2021).

Most of the published evidence on AI-driven irrigation originates from precision agriculture and smart-farming contexts broadly, including horticulture and high-value crops; studies that quantify the specific contribution of AI to cereal water productivity under field conditions remain comparatively few (Sharma et al., 2021; Ali et al., 2023). The manuscript therefore deliberately avoids reporting cross-crop AI performance figures as if they were cereal-specific. Multi-site, multi-season cereal field trials that isolate the contribution of AI from that of the underlying sensor infrastructure are identified here as a priority research need.

The convergence of AI with IoT, often termed AIoT, is emerging as an enabling framework for smart agriculture (Muhammed et al., 2024). Interconnected sensor networks combined with edge and cloud computing can support site-specific monitoring, predictive irrigation scheduling, and, in some applications, anomaly detection for sensor or system faults (Muhammed et al., 2024). More broadly, digital agriculture is increasingly linking irrigation management with nutrient management, pest monitoring, and yield forecasting within integrated decision-support frameworks, although fully unified implementations remain at an early stage (Wolfert et al., 2017). Wider scalability of these approaches in cereal systems, especially in resource-constrained settings, depends on lower hardware and computing costs, improved rural connectivity, interoperable data systems, and user interfaces that are accessible to farmers with limited technical expertise (Klerkx et al., 2019).

### 7.4 Economic feasibility, adoption barriers, and equity considerations

The technical potential of digital irrigation cannot be evaluated in isolation from the socio-economic conditions under which it is deployed. Five sets of constraints are recognized in the literature and treated critically below.

#### 7.4.1 Upfront and operating costs

Representative configurations of IoT-based irrigation systems — soil-moisture probes, a local gateway, a cloud subscription, and a variable-rate application interface — carry upfront hardware costs that are substantial relative to the net revenue of smallholder cereal production. Operating costs include calibration, battery replacement, data subscription, and eventual sensor replacement, typically amortized over 3–5 years. These cost structures have, to date, been economically viable primarily in large commercial operations with high-value crops, and their extension to smallholder cereal systems requires either radical cost reduction or cooperative-ownership business models.

#### 7.4.2 Connectivity

Reliable rural 3G/4G coverage is a necessary condition for most cloud-based irrigation systems, and coverage remains uneven in many sub-Saharan African and South Asian cereal-producing regions. Emerging low-power wide-area network options (LoRaWAN, NB-IoT) and satellite-based connectivity (LEO constellations) are narrowing this gap but do not eliminate it (Elijah et al., 2018).

#### 7.4.3 Technical capacity

Sustained operation of sensor-based irrigation systems requires local capacity to calibrate, troubleshoot, and maintain the equipment. Many pilot deployments have reported high rates of sensor failure and data loss within 2–3 seasons in the absence of a robust extension and after-sales support infrastructure. Capacity-building through extension services, farmer-field schools, and agricultural-technician training is therefore not optional but essential.

#### 7.4.4 Data sovereignty and governance

Most commercial IoT-irrigation platforms store farm-level soil, weather, and yield data on vendor-controlled servers, raising concerns about data ownership, price-discrimination risk, and downstream use for insurance and credit underwriting. Cooperative and public-sector data-governance models are being piloted in several jurisdictions and offer a plausible path to adoption in smallholder contexts, but regulatory clarity on agricultural data rights remains uneven.

#### 7.4.5 Equity risk

There is a tangible risk that digital-irrigation adoption in its current form will entrench rather than reduce existing resource inequalities, because its benefits accrue disproportionately to better-capitalized and better-connected producers. Deliberate equity design — subsidy structures targeted at smallholder cooperatives, service-provider business models that share risk across members, and open-source tools that keep the marginal cost of participation low — is therefore required if digital irrigation is to contribute to rather than detract from broader food-security objectives.

#### 7.4.6 Integration with other levers

Digital-irrigation tools realize their greatest potential value when combined with an agronomic substrate that they can refine (DI, CA, mulching — Sections 4 and 5) and with genetic material whose physiological thresholds they can track (drought-resilient cultivars — Section 6). A sensor network in a field sown with a drought-avoidant cultivar under conservation tillage provides the operator with precise, actionable information; the same sensor network in a conventionally tilled field with a drought-sensitive cultivar provides information that the operator cannot act on without violating the crop’s physiological tolerances. The institutional and governance conditions that determine whether this integration can occur are treated in Section 8.

## 8 Integrated water management: A G×A×D framework

**Figure 2:**
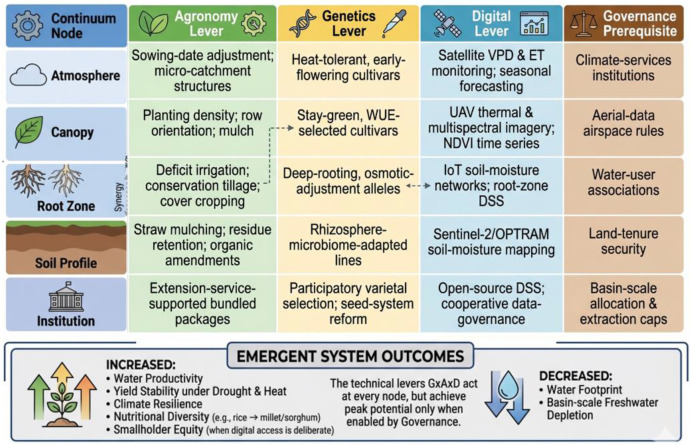
Genetics × Agronomy × Digital (G×A×D) integration framework. The rows describe the soil-plant-atmosphere-institution continuum. The columns detail technical levers (Agronomy, Genetics, Digital) and the overarching Governance prerequisite that enables technical lever combination. Inter-lever synergies are highlighted (dashed arrows), and collectively they drive emergent water-smart system outcomes.

The preceding five sections, considered jointly, point to a consistent conclusion: no single intervention is sufficient, and the largest and most reliable gains in WP and yield stability arise from the co-deployment of complementary agronomic, genetic, and digital levers within a supportive institutional environment. The aggregated effect ranges, principal trade-offs, implementation constraints, and strongest evidence sources for each intervention class are summarized in **Table 2**. This review proposes an explicit Genetics × Agronomy × Digital (G×A×D) integration framework (**Figure 2**) to make that co-deployment operational.

### 8.1 Three illustrative integrated packages

The outcomes cited for each package are expected outcomes, synthesized by combining the independent outcomes reported for each constituent lever in the cited source studies. As noted in Section 9, fully integrated multi-lever field trials of these packages have not yet been conducted and represent a priority evidence gap. The packages should therefore be read as illustrative combinations grounded in published single-lever evidence rather than as reports of documented integrated trials.

Package A — Indo-Gangetic Plains rice–wheat: ZT wheat + AWD rice + decision-support. Zero-till wheat (Erenstein and Laxmi, 2008) combined with mild AWD in rice (Lampayan et al., 2015; Carrijo et al., 2017), drought-tolerant wheat germplasm from CIMMYT pipelines (Singh et al., 2025), and emerging mobile-phone-delivered irrigation advisories illustrates a documented integrated package. Reported outcomes include ~1 irrigation saved per wheat season, 4–6% wheat yield gain, and 23% water savings in rice with no significant yield penalty. The governance prerequisite is a functioning water-user association and the partial relaxation of free-electricity-for-irrigation subsidies that currently blunt incentives for water saving.

Package B — North China Plain wheat–maize: DI + straw mulch + stay-green germplasm. APSIM-based optimization of pre-anthesis DI (Ren et al., 2022), straw strip-mulching (Chai et al., 2022), and adoption of stay-green maize lines illustrate a second integrated package in an intensively groundwater-stressed region. Reported outcomes include stabilized wheat WP under SSP245 climate scenarios, +27% grain yield under straw-mulch substitution for plastic film, and reduced reliance on non-renewable aquifer withdrawal. The governance prerequisite is a functioning groundwater-extraction cap with enforceable metering.

Package C — Semi-arid West African sorghum: stay-green hybrids + ridge-and-furrow + mobile advisory. Deployment of ICRISAT stay-green sorghum hybrids (Borrell et al., 2014), ridge-and-furrow micro-catchment rainfall harvesting (Ren et al., 2017), and mobile-phone-delivered sowing-date and pest advisories illustrates a third package oriented toward rainfed smallholder systems. Reported outcomes include grain yield within 5–8% of well-watered controls under terminal drought and +21% yield under ridge-and-furrow structures. The governance prerequisite is a functional farmer-cooperative seed system and extension infrastructure to support multi-lever adoption.

### 8.2 Climate adaptation, water footprint, and water governance

Water-smart cereal production is closely linked to climate-change adaptation because many strategies that improve WP also enhance resilience to drought and heat stress (IPCC, 2023). Gridded crop-model ensembles driven by SSP climate scenarios indicate that end-of-century maize productivity may decline by about 6% under SSP126 and up to 24% under SSP585, whereas wheat may show net global gains because of CO__2__ fertilization and high-latitude expansion, with substantial regional disparities (Jägermeyr et al., 2021). Multi-model analyses indicate that global wheat yields decline by approximately 6% for each 1 °C rise in temperature, with the most significant reductions occurring in warmer production regions (Asseng et al., 2014). Sowing-date adjustment, improved cultivars, and improved water management will therefore be essential, although their effectiveness diminishes under higher warming levels, reinforcing the need for mitigation alongside adaptation (Jägermeyr et al., 2021; IPCC, 2023).

From a water-governance perspective, improving irrigation efficiency does not automatically reduce total water consumption at the basin scale, because reduced return flows and expansion of irrigated area can offset field-level savings (Ward and Pulido-Velazquez, 2008; Grafton et al., 2018). Ensuring that on-farm efficiency gains translate into real reductions in freshwater abstraction requires water-allocation rules, extraction caps, and monitoring of actual water depletion rather than of withdrawals (Ward and Pulido-Velazquez, 2008; Grafton et al., 2018). Reconciling irrigated food production with environmental-flow requirements at the basin scale is a further layer of this challenge and has been shown to be achievable only when agricultural water demand is bounded by explicit environmental allocations (Jägermeyr et al., 2017). In regions affected by agricultural economic water scarcity—defined as a lack of irrigation due to limited institutional and financial capacity rather than hydrologic constraints—sustainable irrigation expansion could substantially increase food production, feeding up to 840 million additional people without aggravating blue-water scarcity (Rosa et al., 2020). These water-governance considerations are complemented by evidence that coordinated improvements in water and nutrient management could close major cereal yield gaps on existing cropland (Mueller et al., 2012). Investment in underserved rainfed regions may therefore offer a more sustainable pathway than further intensification in already over-exploited basins.

## 9 Future research directions and knowledge gaps

Substantial progress has been made in developing water-smart technologies for cereal production, but the present synthesis identifies seven specific gaps that merit priority attention.

- Long-term DI performance under progressive climate change. Most DI evidence comes from relatively short-term experiments under present-climate variability. Longer-duration field studies and coupled climate–crop model analyses are needed to establish whether current yield-maintaining responses persist under higher baseline temperatures and evaporative demand (Fereres and Soriano, 2007; Ren et al., 2022). Jägermeyr et al. (2021) indicate that negative impacts on global maize production may emerge as early as the 2030s under high-emission scenarios, while global wheat responses remain more heterogeneous, which further tightens the horizon for actionable evidence.
- Genotype-by-management interactions. Cultivar performance depends strongly on the interaction between genotype, management, and environment, yet G×M×E design is under-represented in breeding and agronomic-evaluation programs (Chenu et al., 2018; Sellami et al., 2024). Routine inclusion of multi-location G×M×E trials in public-sector cereal breeding would accelerate the translation of water-saving interventions into farmer-field outcomes.
- Low-cost, connectivity-tolerant digital tools for smallholder systems. The adaptation of smart-irrigation and digital-monitoring technologies to smallholder cereal production requires deliberate design for low cost, robust field operation, and tolerance of intermittent connectivity (Bwambale et al., 2022). This is a design-research priority, not merely a diffusion question.
- Integrated multi-criteria assessment frameworks. Water-smart cereal production should be evaluated not only on yield and WP but also on carbon dynamics, nutrient cycling, biodiversity, and water-quality outcomes (Power, 2010). Assessment frameworks that support trade-off analysis across these criteria are needed to prevent the optimization of WP from inadvertently degrading other ecosystem services.
- Participatory and co-designed research. Ensuring that innovations are locally appropriate, socially acceptable, and economically viable requires participatory and co-designed research approaches (Van de Fliert and Braun, 2002). The present review identifies participatory varietal selection and cooperative data-governance models as particularly promising loci for co-design.
- Green-water management. Improved retention of rainfall in the soil profile (green water) could avert crop losses sufficient to feed approximately 670 million people under 3 °C warming (He and Rosa, 2023), yet green-water management receives far less attention than irrigation-focused blue-water strategies. Explicit mainstreaming of green-water metrics in water-smart policy frameworks is overdue.
- Integrated G×A×D package field evidence. Most of the evidence reviewed here addresses single-lever or two-lever combinations; multi-location, multi-season field trials of fully integrated G×A×D packages (as illustrated in Section 8.1) are essentially absent from the cereal literature. This is the single largest methodological gap identified by the present review and is the most direct route to rigorous, actionable evidence on water-smart cereal production.

## 10 Conclusions

The transition toward water-smart cereal production is becoming a central requirement for sustaining food security under increasing water scarcity and climate risk. This narrative review synthesized 110 studies across agronomic, genetic, and digital innovations and proposed the Genetics × Agronomy × Digital (G×A×D) framework as an operational map of how these three intervention families can be combined along the soil–plant–atmosphere–institution continuum.

The binding constraint on water-smart cereal production has shifted. It is no longer the technical feasibility of individual interventions — the portfolio of deficit irrigation, alternate wetting and drying, straw and film mulching, conservation agriculture, stay-green and drought-tolerant cultivars, and IoT-based scheduling is broad, mature, and supported by meta-analytic evidence in its major components. The binding constraint is now the institutional, financial, and governance capacity required to combine these interventions equitably across the world’s approximately 730 million hectares of cereal-producing land. Technical investment in single-lever optimization will yield diminishing returns unless it is matched by investment in seed systems, extension services, cooperative data governance, and basin-scale water-allocation reform.

Three priorities for the next five years: (1) multi-location, multi-season field trials of fully integrated G×A×D packages in the three agroecological contexts illustrated in Section 8.1 (Indo-Gangetic Plains rice–wheat; North China Plain wheat–maize; semi-arid West African sorghum), with explicit cost–benefit and equity components. (2) Design-oriented research on low-cost, connectivity-tolerant digital tools tailored to smallholder cereal systems, replacing the prevailing extrapolation from high-value horticultural prototypes. (3) Water-governance reforms — extraction caps, allocation instruments, cooperative data regimes — that convert on-farm efficiency gains into basin-scale water savings and prevent the paradox of irrigation efficiency from offsetting technical progress.

As the global population approaches approximately 9.7 billion by 2050, water-smart cereal production offers credible pathways for improving output under tightening resource constraints, but only if technical, institutional, and governance innovation proceed together. The G×A×D framework proposed here is intended to make that combined agenda explicit, operational, and measurable.
